# Influence of short-fiber composite base on fracture behavior of direct and indirect restorations

**DOI:** 10.1007/s00784-020-03768-6

**Published:** 2021-01-08

**Authors:** S. Garoushi, S. Sungur, Y. Boz, P. Ozkan, P. K. Vallittu, S. Uctasli, L. Lassila

**Affiliations:** 1 Department of Biomaterials Science and Turku Clinical Biomaterial Center-TCBC, Institute of Dentistry, University of Turku, Turku, Finland; 2 Department of Prosthodontics, Faculty of Dentistry, University of Ankara, Ankara, Turkey; 3 City of Turku Welfare Division, Oral Health Care, Turku, Finland

**Keywords:** CAD/CAM, Bi-structure restoration, Overlays, Short-fiber composite

## Abstract

**Objectives:**

The aim was to examine the influence of short-fiber composite (SFC) core on the fracture-behavior of different types of indirect posterior restorations. In addition, the effect of thickness ratio of SFC-core to the thickness of the veneering conventional composite (PFC) on fracture-behavior of bi-structured composite restorations was evaluated.

**Materials and methods:**

MOD cavities with removed palatal cusps were prepared on 90 intact molars. Five groups of direct overlay restorations (*n* = 10/group) were fabricated having a SFC-core (everX Flow) with various thicknesses (0, 1, 2, 3, 4 mm) and layer of surface PFC (G-aenial Anterior), remaining the thickness of the bi-structure restoration to be 5 mm. Four groups of CAD/CAM-made restorations (Cerasmart 270 and e-max CAD) were fabricated either with 2-mm layer of SFC-core or without fiber reinforcement. Intact teeth (*n* = 10) were used as control group. Restorations were statically loaded until fracture. Fracture patterns were evaluated visually. Data were analyzed using ANOVA (*p* = 0.05).

**Results:**

With indirect overlay restorations, no statistically significant differences (*p* > 0.05) were observed in the load-bearing capacities between restorations reinforced by 2-mm SFC-core (bi-structured) and those fabricated from plain restorative materials. ANOVA displayed that direct overlay restorations made from 4-mm layer thickness of SFC-core had significantly higher load-bearing capacities (3050 ± 574 N) (*p* < 0.05) among all the groups tested.

**Conclusions:**

Restorations (direct/indirect) combining SFC-core and a surface layer of conventional material demonstrated encouraging achievement in reference to fracture behavior.

**Clinical relevance:**

The use of flowable short-fiber composite as reinforcing base with large direct and indirect restorations may result in more repairable failure.

## Introduction

Several treatment options are available to restore posterior teeth with severe coronal damages, representing a daily challenge in clinical practice. Routine use of full crown restorations applies the removal of remaining sound tooth structure. As an alternative, adhesively cemented ceramic overlay restoration has been used with a view to minimize the removal of remaining tooth structure. Along with modern materials, there has been a simultaneous development in fabrication techniques for ceramic restorations. There has been a shift from the traditional hand-layering technique to computer-aided design computer-aided manufacturing (CAD/CAM) technology [1]. However, full ceramic restorations have got some shortcomings. They are brittle and very costly, request more tooth reduction, may provoke abrasive wear of antagonist teeth, and need longer chairside time because of their challenging bonding procedure [2, 3]. An affordable alternative to full ceramic restorations is identified in resin composite restorations. In contrast to ceramics, resin composites are cheaper, simple to use, and do not induce wear of the opposing teeth [4–6]. Numerous investigations were performed on the practical efficiency of resin composite restorations made by either direct manual buildup technique or the CAD/CAM technique [7–9]. The most common cause for failure in all trials was fracture, indicating that the fracture toughness of resin composite restorations is among the most significant characteristics in order to achieve satisfactory clinical outcomes. According to literature, particulate-filled resin composite (PFC) materials yet show hindrance because of their inadequate toughness when applied in high-stress bearing areas [3, 10]. Owing to this kind of failures, it is still uncertain, whether PFCs has to be applied in high-stress bearing applications like posterior overlay or large MOD (mesio-occlusal-distal) restorations. A lot of research has been performed in order to develop a technique to reinforce the large composite restorations and the remaining tooth structure. One of those advancements in resin composite technology to support its use in complex clinical situations is the evolution of short-fiber-reinforced composite (SFC) material where the filler system is potentiated with short glass fibers to resist crack propagation [10–13]. The attempt was to use SFC as supporting core under veneer or surface layer of PFC material, which could be judged as bi-structured composite restorations [14, 15]. Several in vitro investigations have proved that teeth restored with a bi-structured system of using SFC as bulk core had higher load-bearing capacity and a favorable fracture pattern [16–19]. They proved that SFC reinforces the remaining tooth structure and composite restoration by serving as a crack-preventing layer [17, 18]. However, to the author’s knowledge, the effect of using SFC as reinforcing core under CAD/CAM-fabricated restorations has not been well investigated. Even though many things is known about the characteristics of SFC or veneering material itself [20, 21], little data exists on the loading performance of material combination (i.e., bi-structured restoration). It can be hypothesized that there are differences in load-bearing capacity and fracture behavior when the volume ratio of SFC to veneering material is changed.

Therefore, the goal of the present research was to examine the influence of SFC-core on the fracture behavior of different direct/indirect posterior overlay restorations. Moreover, the effect of thickness ratio of SFC-core to the thickness of the veneering PFC on fracture behavior of bi-structured composite restorations was evaluated.

## Materials and methods

The materials used in this study with their composition are listed in Table 1.

**Table 1:** The restorative materials used in the study

Material (code)	Manufacturer	Composition
G-aenial Anterior (PFC)	GC Corp, Tokyo, Japan	UDMA, dimethacrylate co-monomers, prepolymerized silica, and strontium fluoride containing fillers 76 wt%
Cerasmart 270	GC Corp, Tokyo, Japan	Bis-MEPP, UDMA, DMA, Silica (20 nm), barium glass (300 nm) 71 wt%
e-max CAD	IvoclarVivadent AG, Liechtenstein	Lithium disilicate glass ceramic
everX Flow (SFC) Bulk shade	GC Corp, Tokyo, Japan	Bis-EMA, TEGDMA, UDMA, short glass fiber (200–300 μm and Ø7 μm), barium glass 70 wt%

*TEGDMA*, triethylene glycol dimethacrylate; *UDMA*, urethane dimethacrylate; *Bis-MEPP*, bis (p-methacryloxy (ethoxy)1-2 phenyl)-propane; *Bis-EMA*, ethoxylated bisphenol-A-dimethacrylate; *wt%*, weight percentage

One hundred extracted, sound, and caries-free mandibular molar teeth of similar occlusal size (± 1 mm) were selected. Upon collection, adhering soft tissues were removed under running water and the teeth were stored in a 0.5% chloramine T solution at 4 °C for a period not exceeding 2 months. The size of each tooth was measured from buccolingual and mesiodistal directions with a digital caliper (Mitutoyo Corp., Tokyo, Japan). The mean dimensions were 10.3 (± 0.5) in buccolingual and 11.3 (± 0.6) in mesiodistal directions. The teeth were mounted on an acrylic block (diameter 2.5 cm) at the cement-enamel junction using auto-polymerized acrylic resin (Palapress; Heraus Kulzer, Wehrheim, Germany). Ninety teeth received a similar coronal preparation. Two operators performed all teeth preparations and restorations. Ten teeth were left intact and served as control.

### Tooth preparation and restorative procedures

**Fig. 1:**
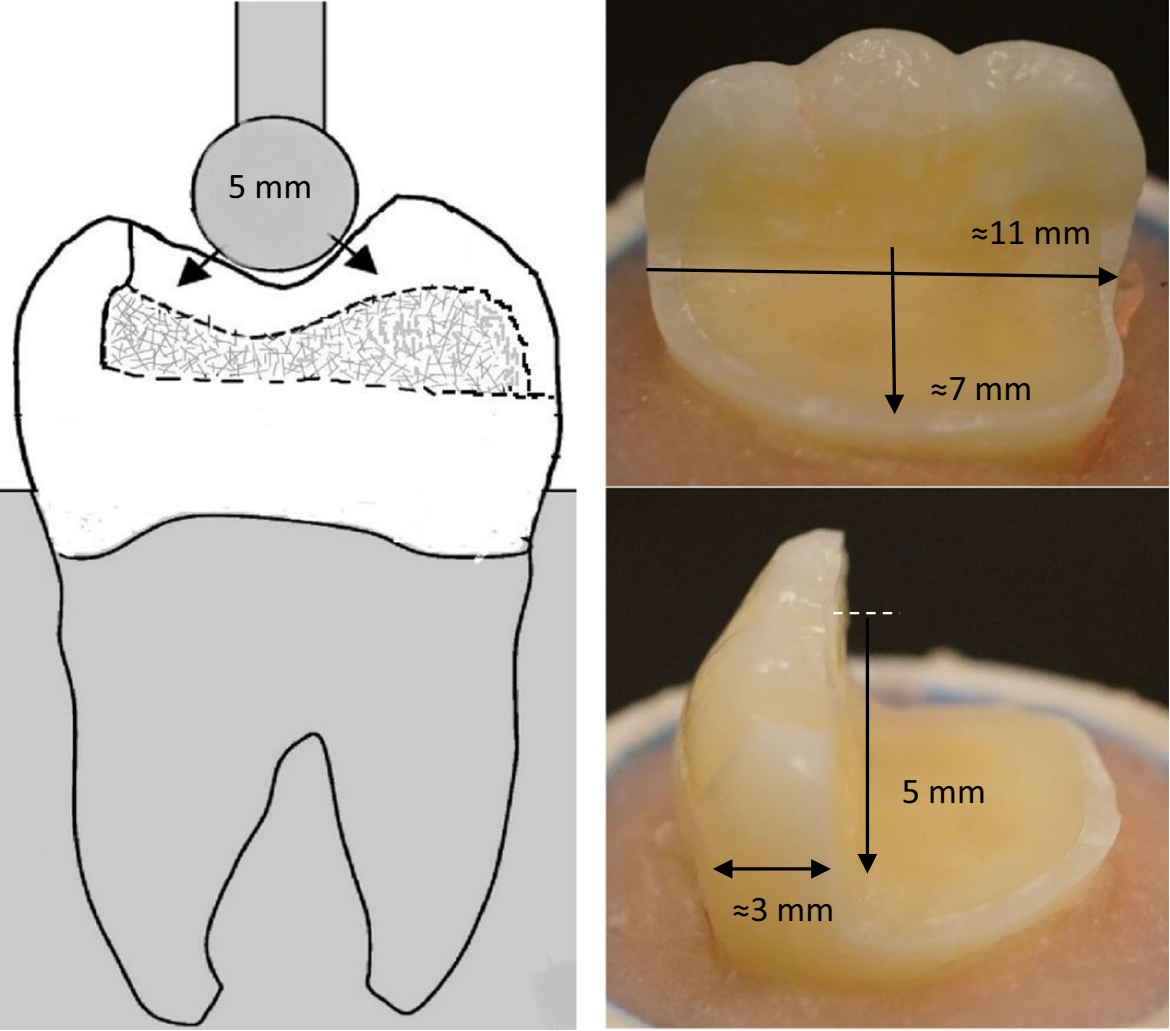
A photograph and schematic drawing representing tooth preparation measurements in millimeters, bi-structured restoration, and the compression load test setup

MOD cavities with removed palatal cusp preparations were fabricated on ninety mandibular molars. The removed palatal cusps were at the level of the isthmus floor. The preparation was made having a flat cavity floor with 5 mm of occlusal reduction (Fig. 1). The remaining buccal wall thickness was around 3 mm. The margins were placed 1–1.5 mm above the cement-enamel junction (CEJ). Preparation was achieved with flat-end parallel carbide bur (H21LR.314.010, Brasseler, Savannah, GA, USA) and round-end diamond bur (850–014 M SSWhite, Lakewood, NJ, USA) at high speed and under water cooling. According to Bijelic-Danova et al., this MOD preparation was named as flat-box type of preparation and represented a situation that was commonly seen after removal of an old complex amalgam restoration [18].

After completing the cavity preparation, the tooth surfaces were prepared for bonding with a one-step adhesive system (G-Premio Bond, GC Corp., Tokyo, Japan) according to the manufacturer instructions. The teeth were then restored via two approaches as direct and indirect restorations for the purpose of imitate clinical techniques.

### Direct restoration

For these groups, a translucent model (Memosil 2, Heraeus Kulzer GmbH, Hanau, Germany) of tooth crown before preparation was used to assist standardized restoration fabrication. In order to study the influence of thickness ratio of SFC-core to the thickness of the veneering PFC (occlusally), more groups were constructed having a SFC-core with various thicknesses (0, 1, 2, 3, 4 mm), remaining the thickness of the bi-structure restoration being 5 mm. The thickness of SFC-core before polymerization was controlled by the use of scaled periodontal probe, as the material was horizontally applied on the flat cavity floor.

- Group 1: 0-mm SFC-core + 5-mm PFC
- Group 2: 1-mm SFC-core + 4-mm PFC
- Group 3: 2-mm SFC-core + 3-mm PFC
- Group 4: 3-mm SFC-core + 2-mm PFC
- Group 5: 4-mm SFC-core + 1-mm PFC

Direct composite restorations were manually made by buildup of PFC (G-aenial Anterior). The PFC pastes were packed into the space created between the index and the prepared cavity (with or without SFR-core), followed by curing through a hand-light curing unit (Elipar TM S10, 3M ESPE, Seefeld, Germany) from all directions and for 40 s per increment (wavelength of the light was between 430 and 480 nm and light intensity was 1600 mW/cm^2^). The light curing tip was in close contact (1–2 mm) with the resin composite surface. The missing axial walls for all groups (bi-structure) were built up with PFC composite (1 mm).

### Indirect restoration

- Group 6: 5-mm Cerasmart 270
- Group 7: 2-mm SFC-core + 3-mm Cerasmart 270
- Group 8: 5-mm e-max CAD
- Group 9: 2-mm SFC-core + 3-mm e-max CAD

Groups (bi-structure) made of SFC as core material (2 mm) leave a space (3 mm occlusally; 1 mm proximally and lingually) for the veneering materials (manual buildup and CAD/CAM-fabricated) to be extended over the whole restoration surfaces.

For CAD/CAM-fabricated groups, a photoimpression of the prepared cavity (with or without SFR-core) was taken, and then, restoration was designed and milled (CEREC, Sirona Dental Systems Inc., Long Island City, NY) of Cerasmart 270 and e-max CAD blocks. Before cementation, the inner surface of all restorations was acid-etched by 9.6% hydrofluoric acid (Pulpdent Corporation, Watertown, MA, USA) for 60 s followed by washing and air-drying. The CAD/CAM-fabricated restorations were then cemented using a multi primer (G-Multi Primer, GC, Tokyo, Japan) and dual-cure resin cement (G-CEM linkForce, GC, Tokyo, Japan), followed by light curing from all directions using a hand-light curing unit (Elipar TM S10) for 20 s per segment. The light source was placed in close contact with the crown surface.

Prior to testing, all restorations were polished using abrasive polishing points (Jiffy Polishers, Ultradent, South Jordan, UT, USA) and stored in water for 48 h at 37 °C.

### Fracture load test

A static compressive load was applied to the restored teeth with a universal testing machine (Lloyd model LRX, Lloyd Instruments Ltd., Fareham, UK) at a speed of 1 mm/min. The loading was applied vertically between the triangular ridge of the buccal and lingual cusps (Fig. 1) using a round-shaped metallic tip (Ø 5 mm). The loading event was registered until restoration fracture (final drop in the load-deflection curve). Fracture patterns of each loaded restorations were visually examined and classified to three typical behavior: catastrophic fracture of restoration and tooth structure, fracture of only restoration, and chipping or delamination of veneered restorative material from SFC-core.

### Microscopic analysis

The representative fractured restorations were selected and examined by scanning electron microscopy (SEM, JSM 5500, Jeol Ltd., Tokyo, Japan). Prior to observation, all the specimens were cleaned by alcohol and then coated with a gold layer using a sputter coater in vacuum evaporator (BAL-TEC SCD 050 Sputter Coater, Balzers, Liechtenstein). The analysis was started from the edge of the fractured restoration specimen, from the upper loading part to the inner surface and ending at the SFC-core.

### Statistical analysis

The data were statistically analyzed with SPSS version 23 (SPSS, IBM Corp.) using analysis of variance (ANOVA) at the *p* < 0.05 significance level followed by a Tukey HSD post hoc test to determine the differences between the groups.

## Results

**Fig. 2:**
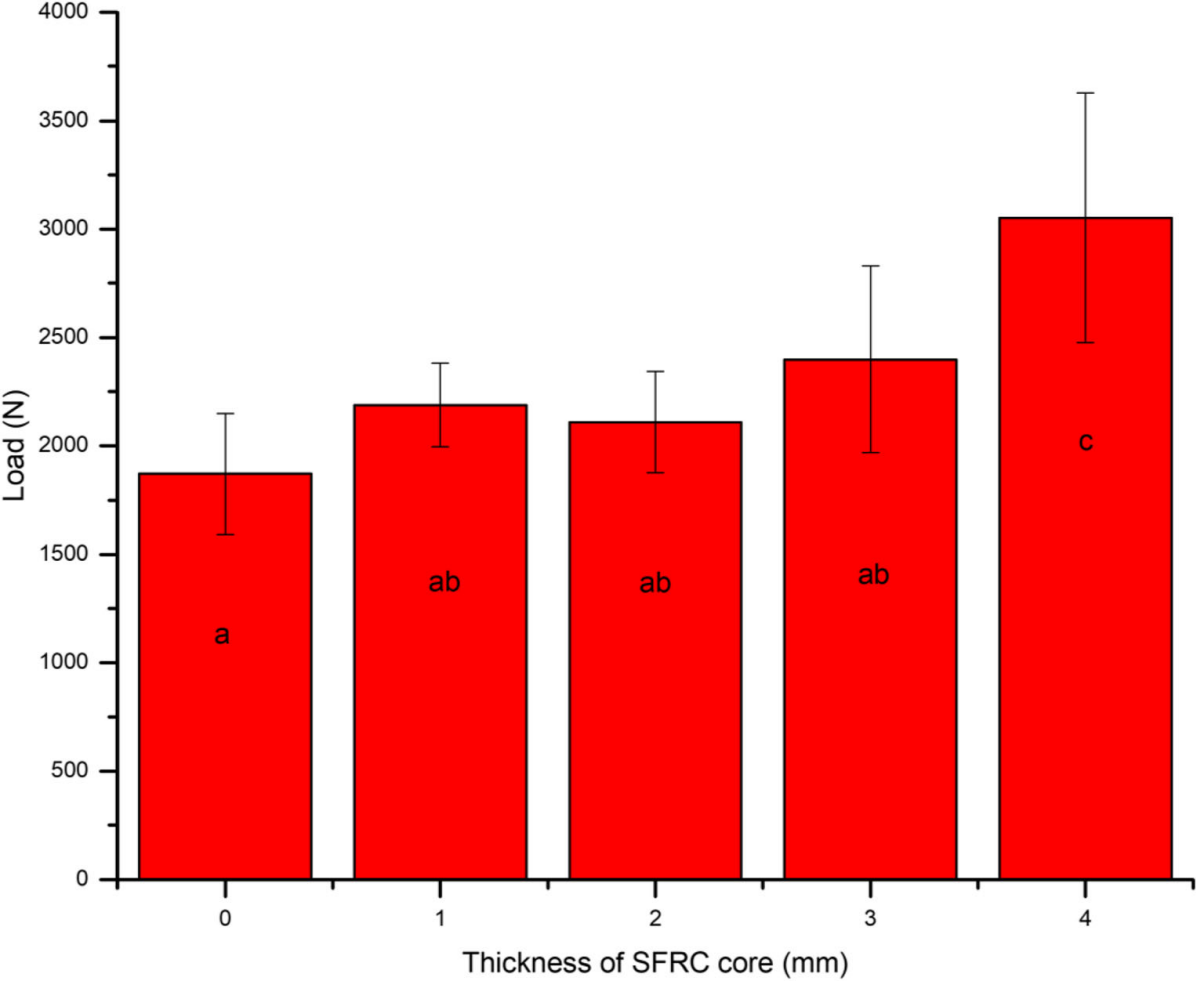
Mean fracture load values (*N*) and standard deviations (SD) of tested composite restorations with different SFC-core thicknesses. The same letters inside the bars represent non-statistically significant differences (*p* > 0.05) among the materials

**Fig. 3:**
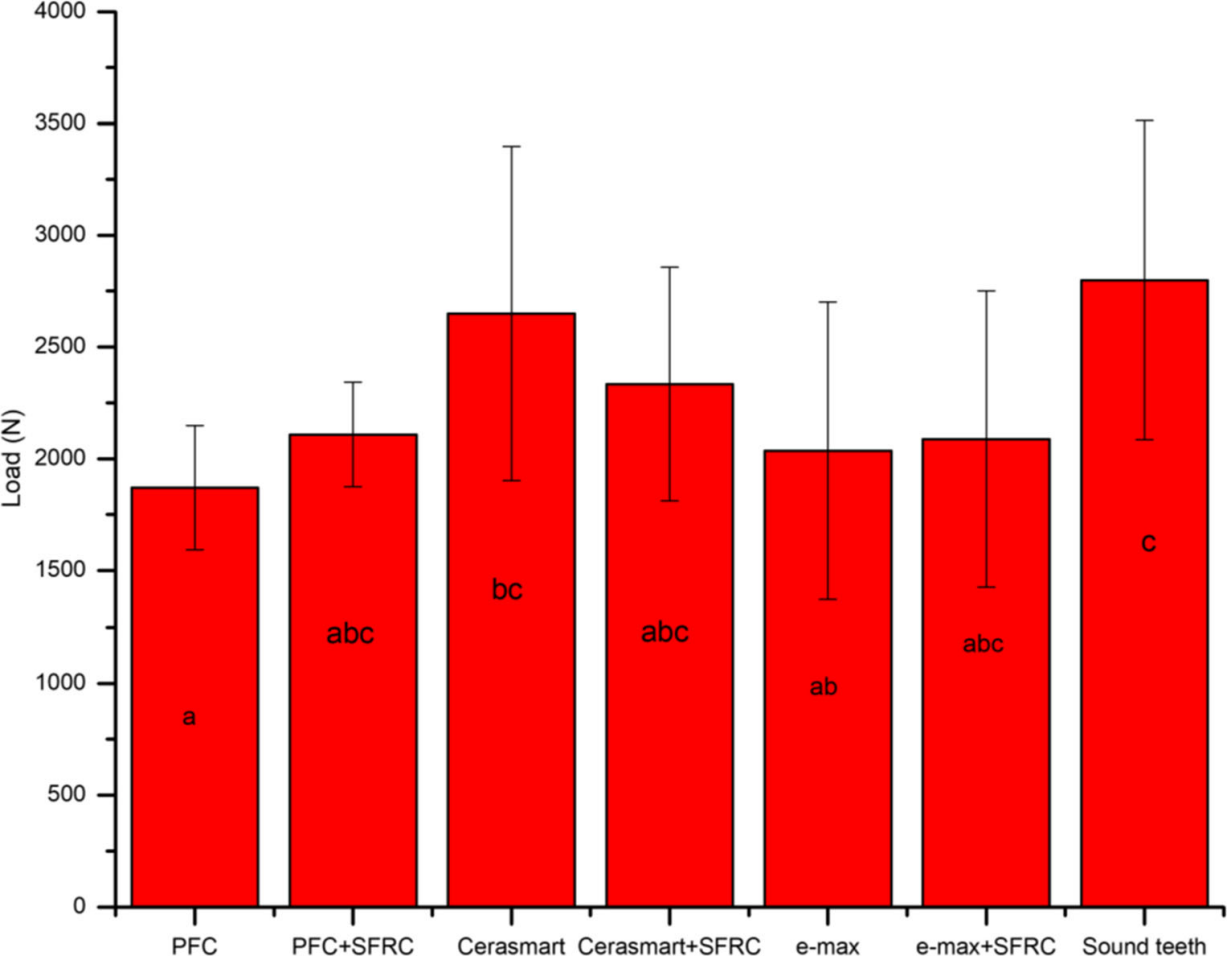
Mean values of load-bearing capacity (*N*) and standard deviation (SD) of tested restorations (single/bi-structure). The same letters inside the bars represent non-statistically significant differences (*p* > 0.05) among the materials

The mean load-bearing capacities of the restorations with different thickness ratios of SFC-core to the veneering PFC are presented in Fig. 2. ANOVA revealed that restorations reinforced by thick SFC-core (4 mm) had significantly higher load-bearing capacities (3051 ± 574 N) (*p* < 0.05) among all tested direct composite restorations. The load-bearing capacity of teeth restored with different techniques (direct/indirect) and reinforced by only 2-mm layer of SFC-core is shown in Fig. 3. No statistically significant differences (*p* > 0.05) were identified in the load-bearing capacities between restorations reinforced by 2-mm SFC-core (bi-structured) and those fabricated from plain restorative materials (single structured).

**Fig. 4:**
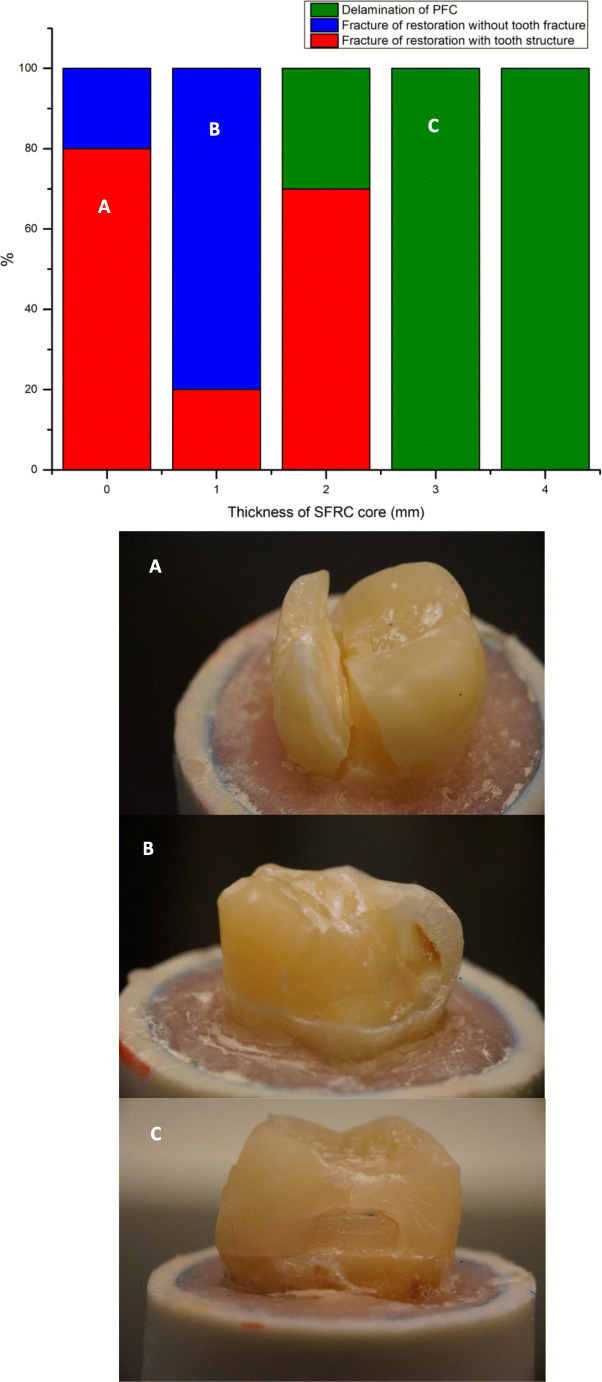
Percentage and photographs of various fracture patterns of the composite restorations with different SFC-core thicknesses

**Fig. 5:**
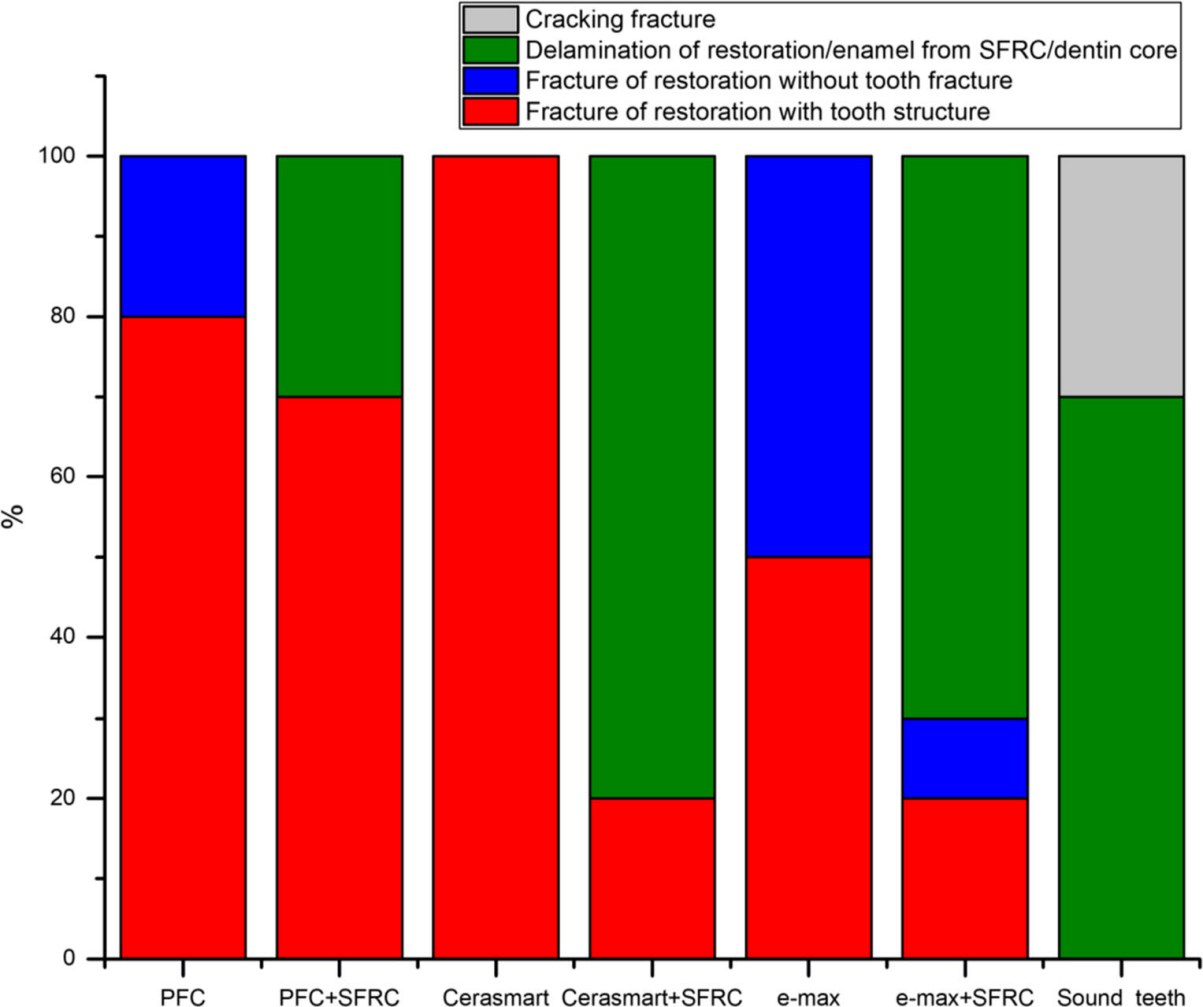
Percentage of various fracture patterns of tested single-structure and bi-structure restorations

**Fig. 6:**
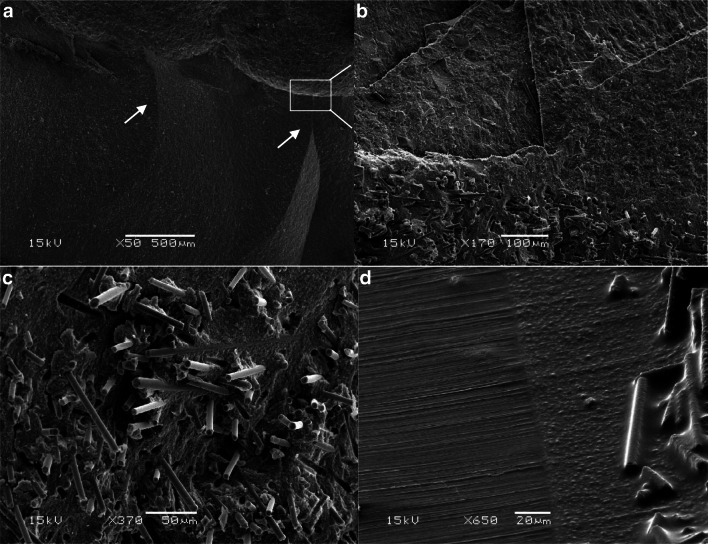
SEM photomicrographs with different magnifications of fracture surfaces of investigated bi-structure restorations showing a radial cracks (**a** and **b**, arrow) propagated from the load application area to the interface at SFC-core. **c** Delamination of veneering material from SFC-core. **d** Interface between veneering material and SFC-core

Regarding fracture pattern, restoration specimens having only conventional PFC veneering material (single-structure) without SFC-core reinforcement revealed more a catastrophic unrepairable fracture pattern (Fig. 4a). Whereas, restoration specimens that had a thick (3 and 4 mm) reinforced core material of SFC showed only chipping of veneered PFC material (repairable fracture) from the SFC-core layer (Fig. 4c). For indirect restorations, restored groups using SFC-core, just as the natural teeth, showed dominantly repairable chipping fractures (Fig. 5). Representative SEM images of fractured bi-structured restorations are shown in Fig. 6.

## Discussion

The present research effort evaluated the effect of two restorative techniques (single-structure or bi-structure with SFC-core) using different direct/indirect restorative materials for huge MOD restorations and their influences on the fracture behavior. The used preparation and restorative outline simulated the circumstances of serious loss of tooth structure that could be restored either directly or indirectly [18]. Our hypotheses are partially accepted, as there was no significant difference found in load-bearing capacity performance among the restorative techniques used (Fig. 3). However, there were differences in fracture pattern (Fig. 5).

This is in compliance with earlier investigations which stated that the inclusion of SFC as base within the cavity of molars and premolars rebuild with thick overlays was not beneficial in enhancing their load-bearing capacity [15, 22–24]. On the contrary, many other investigations have indicated that posterior teeth restored with a bi-structured composite technique of using SFC as a bulk core material had superior load-bearing capacity and a favorable fracture pattern [25–27]. Such differences among studies could be derived from the difference in the thickness ratio between the SFC-core composite and overlay material, the test setup, and the bonding technique used.

Because the current composite and ceramic materials are brittle, they do not lack strength, but they require toughness [28]. One of the main drawbacks of brittle materials when employed to replace the lost dentin is the substantially lower fracture toughness of these materials in comparison with that of the dentin [11]. The concern of lack of fracture toughness is clearly observed in large restorations, as the volume of the brittle material increases [29]. As a consequence of the above-described shortcoming, direct/indirect composite restorations probably not the ideal option in a situation of significant loss of tooth structure.

As already mentioned, fracture toughness property characterizes the resistance of brittle materials to the crack propagation under an applied load [10]. Therefore, it explains fracture resistance of the material and could be considered a scale of fatigue durability, which predicts structural performance. The new flowable SFC (everX Flow) used in this investigation has earlier been informed to display high flexural strength and fracture toughness [16, 20, 30]. As far as we know, there were no other dental composites with fracture toughness values around 2.6 MPa m^1/2^. Existing data with regard to fracture toughness values of various direct/indirect restorative materials like composite and ceramic are in range of 1.1 to 1.9 MPam^1/2^ [31, 32].

Restoration specimens having only veneering material without any fiber reinforcement displayed more catastrophic unrepairable fracture pattern (Figs. 4 and 5). As reported by Chai, this appears to be median-radial cracks expanding from the loading point into the material [33], clearly demonstrating that the brittleness of the veneering material generated the catastrophic unrepairable fracture. On the other side, all of the restorations that have SFC-core revealed dominantly chipping of veneering material from the reinforcing core. Thereby, the fracture pattern shifted to mainly repairable fractures, compared to the plain veneering material restoration groups (single-structured). Curiously, such chipping fracture pattern was similar to natural crown fracture patterns observed in this and earlier studies [10].

Our data showed substantial improvements in the fracture behavior of the restorations when a thick SFC-core was used compared to that of plain PFC (Fig. 2). The role of SFC-core is predicated on supporting the PFC layer and acting as a crack-stopping layer [34]. To get reinforcement from the SFC-core for the PFC, the integral toughness of the SFC-core should be superior than that of the PFC surface layer [16, 35]. In view of this, the fiber orientation and the polymer matrix cross-linking density probably have a major role. From another point of view, if the role of the SFC-core is based on the mechanism of a crack-stopper, the distance from the surface of the stress initiation point to the SFC-core is of importance. Thus, the veneered PFC thickness might contribute to the load-bearing capacity and crack propagation. This is consistent with earlier investigations which showed the importance of how thick SFC and PFC layers should be applied [35–37].

Regardless to the high applied forces (above the masticatory force), none of the restorations (direct/indirect) failed adhesively, which reflects to the sufficient level of obtained bonding. The adhesion between the CAD/CAM material and the luting cement could be due to the combination of chemical bonding with the use of primer and micromechanical retention aided by acid etching.

The load-bearing values determined by many researchers were reported under various parameters. These parameters were either absolute reduction in the load amount or initial cracking that was interpreted as crack development. In the present study, the utmost loading force on the final fracture was determined. Stresses applied to dental restorations and teeth are usually cyclic and low rather than being impactive in nature. However, because of a relative relationship between static and fatigue loading, the impactive static test would also provide relevant data regarding the fracture behavior and load-bearing capacity [25, 36]. Another limitation of this study is that the periodontal ligament was not simulated. The periodontal ligament mimics the physiological tooth mobility and effect on the fracture occurrence as previous research showed that omitting the artificial periodontium during loading test caused fracture results almost twice the fracture force compared to tests with periodontium [29, 38, 39].

From practical point of view, it is certain that morphology and occlusion are best controlled with indirect overlay restorations instead of direct techniques. However, economic efficiency of the patient might be restricted. Clinical trials should be conducted to confirm the usefulness of bi-structured restorations using the SFC-core as a dentin substitute.

## Conclusion

Within the limitations of this study, the following conclusions can be drawn:

1. Large MOD direct/indirect restorations combining thick SFC-core and a surface layer of veneering material, demonstrated encouraging achievement in reference to fracture behavior.
2. Indirect single-structure composite restoration for large MOD cavities displayed better performance related to fracture behavior than single-structure direct composite restoration.
